# Phosphonate-driven oxic CH_4_ production by *Variovorax xiamenensis* sp. nov.: insights from marine and freshwater microbial adaptations to P-limitation

**DOI:** 10.1093/ismeco/ycaf100

**Published:** 2025-06-16

**Authors:** Yu Wang, Shaohe Wang, Zifu Xu, Silin Ni, Min Xu, Shuh-Ji Kao

**Affiliations:** State Key Laboratory of Marine Environmental Science and College of Ocean and Earth Sciences, Xiamen University, Xiamen, Fujian 361102, China; State Key Laboratory of Marine Environmental Science and College of Ocean and Earth Sciences, Xiamen University, Xiamen, Fujian 361102, China; State Key Laboratory of Marine Resource Utilization in South China Sea, School of Marine Science and Engineering, Hainan University, Haikou, Hainan 570228, China; State Key Laboratory of Marine Resource Utilization in South China Sea, School of Marine Science and Engineering, Hainan University, Haikou, Hainan 570228, China; State Key Laboratory of Marine Resource Utilization in South China Sea, School of Marine Science and Engineering, Hainan University, Haikou, Hainan 570228, China; State Key Laboratory of Marine Resource Utilization in South China Sea, School of Marine Science and Engineering, Hainan University, Haikou, Hainan 570228, China

**Keywords:** methylphosphonate (MPn) catabolism, heterotrophic bacteria, global warming, phosphorus cycling, methane emissions

## Abstract

Methane (CH₄) emissions in oxygenated aquatic environments challenge traditional views of methanogenesis suggesting the presence of alternative microbial CH₄ production pathways. This study identifies a novel bacterial species, *Variovorax xiamenensis* W6^T^, capable of utilizing methylphosphonate (MPn) as its sole phosphorus (P) source under laboratory conditions, thereby supporting growth and CH₄ production in phosphorus-limited conditions. Transcriptomic analyses, as well as incubation experiments, reveal that CH₄ emission via the phosphonate-degrading *phn* gene cluster, encoding a C–P lyase, is tightly regulated by inorganic phosphate (Pi) availability, linking nutrient scarcity to methane cycling. A genomic survey of over 16 000 prokaryotic genomes reveals the widespread occurrence of *phn* gene cluster in 9% of the analyzed genomes, predominantly within the phylum *Pseudomonadota*. While MPn metabolism has been well-documented in marine environments, its presence and ecological role in freshwater systems remain underexplored. Our findings highlight the potential for phosphonate-driven methane production in freshwater ecosystems and underscore the need for further research to quantify MPn concentrations and its contribution to global methane budgets. This study emphasizes the importance of integrating microbial phosphonate metabolism into models of biogeochemical cycling and climate predictions, particularly under scenarios of increasing phosphorus limitation driven by global warming.

## Introduction

Global warming is expected to prolong thermal stratification in aquatic systems [1–3], exacerbating nutrient depletion in surface waters due to reduced mixing with deeper, nutrient-enriched waters [4–6]. In many deep lakes and reservoirs, this phenomenon, combined with increasing bioavailable nitrogen inputs [7], has intensified phosphorus (P) limitation [8, 9]. Under such P-deficient conditions, microbial communities have evolved specialized adaptations to access alternative phosphorus sources [10].

One well-documented adaptation is the expression of the high-affinity phosphate transport system encoded by the *pstABCS* operon [11], enabling efficient scavenging of inorganic phosphate (Pi) under limiting conditions. Additionally, microbial communities may utilize dissolved organic phosphorus (DOP) [12] such as phosphomonoesters and phosphonates [13], which dominate the dissolved organic phosphate pool in some environments [14]. A phosphonate-degrading *phn* gene cluster, along with substrate-specific pathways (*palA*, *phnA*, *phnW*, *phnX*, *phnY*, *phnZ*) enables some microbes to cope better with P-limited conditions by using phosphonates as a source of phosphorus [15]. The *phn* gene cluster consists of a phosphonate transporter complex (*phnC-E*), a transcriptional regulator gene (*phnF*), a core function C–P lyase complex (*phnG-M*) and several accessory genes (*phnN-P*) [16]. The identification of *phn* gene cluster within globally important marine bacterial lineages, such as SAR11 [17], provides insights into the mechanisms by which Marine methane paradox these organisms adapt to phosphate-limited environments.

Among biogenic phosphonates, methylphosphonate (MPn) stands out due to its widespread microbial origin in marine systems [18–20] and its role in oxic methane production (OMP) [21–23]. In marine environments, MPn degradation has been recognized as a significant factor driving methane supersaturation in oligotrophic regions, such as the North Pacific [22]. Recent studies have shown that heterotrophic bacteria harboring the C–P lyase gene cluster, a key component of the *phn* gene cluster, are abundant and their activities are linked to methane accumulation in oxygenated waters [24]. Furthermore, diverse cyanobacteria possessing the full *phn* gene cluster [25] have been implicated in methane supersaturation in the epipelagic zone of marine ecosystems [26], underscoring the profound implications of MPn degradation for both phosphorus cycling and methane emissions.

While MPn metabolism has been extensively studied in marine environments, its ecological significance in freshwater systems is increasingly recognized, with recent studies [23, 27–29] providing valuable insights into its role in these ecosystems. This study presents a comprehensive phenotypic and genomic analysis of *Variovorax xiamenensis* W6^T^, a novel species within the genus *Variovorax*, with a focus on its ability to utilize MPn as a sole phosphorus source and its methane production potential under laboratory conditions. RNA-seq analysis was employed to further elucidate the transcriptional response of W6^T^ to MPn degradation. To explore the prevalence and distribution of the *phn* gene cluster across diverse habitats, over 16 000 prokaryotic genomes from the ProGenomes3 database were analyzed. These findings not only deepen the understanding of microbial MPn utilization but also highlight its potential implications for methane emissions and phosphorus cycling in freshwater ecosystems, pending further validation through field studies.

## Materials and methods

### Bacterial isolation and characterization

The strain W6^T^ was isolated from a freshwater sample collected from the surface of Shidou Reservoir in Xiamen, Fujian Province, China (24.685°N, 118.0139°E) in April 2022. The sample was serially diluted and plated on Luria-Bertani Agar (LB, BD) and incubated at a 28°C for one week, resulting in the isolation of a yellow colony designated as W6^T^. Cell morphology and size were examined using transmission electron microscopy (HT-7800; Hitachi) after negative staining with 2.0% (w/v) uranyl acetate. Gram staining was conducted as described by Hucher [30]. Anaerobic growth was tested according to the procedure of Dong and Cai [31]. Catalase and oxidase activities were tested using 3% (v/v) H_2_O_2_ and 1% (w/v) *N*,*N*,*N′*,*N′*-tetramethyl-1,4-phenylenediamine, respectively. Growth parameters were tested in LB at temperatures ranging from 4°C to 45°C and pH levels from 4.0 to 10.0, using appropriate biological buffers, including citrate/phosphate (pH 4.0–7.0), Tris/HCl (pH 8.0–9.0), and sodium carbonate/sodium bicarbonate (pH 10.0). Salinity tolerance was evaluated with NaCl concentrations ranging from 0% to 7% (w/v). Carbon source utilization, acid production and additional physiological and biochemical characteristics were assessed using the API 20NE and API ZYM systems (bioMerieux, France). Cellular fatty acid profiles of strain W6^T^ were processed using the standard MIDI protocol, analyzed via gas chromatography (Agilent Technologies), and identified using the TSBA6.0 database [32]. Respiratory quinone and polar lipids were analysed by the methods described previously [33, 34].

### Phylogenetic and genome sequence analyses

The 16S rRNA gene of strain W6^T^ was amplified using colony PCR with the universal primers 27F and 1492R [35]. Sequence similarity was determined using the GenBank database and EzBioCloud server [36]. Phylogenetic analysis was conducted using MEGA v11 software [37] with the Kimura two-parameter model and clustering with the neighbor-joining [38], maximum likelihood [39] and minimum evolution [40] methods. Bootstrap values were calculated based on 1000 replications to support the phylogenetic tree [41]. The genome of strain W6^T^ was sequenced using the Illumina Hiseq X10 platform (PE 150 mode) by Shanghai Majorbio Bio-Pharm Biotechnology. Clean reads were obtained by filtering out low-quality bases and assembled using SOAPdenovo2 software [42]. Comparative genomic analyses between strain W6^T^ and reference strains were conducted using multiple metrics: average nucleotide identity (ANI) via EzGenome web service [43], digital DNA–DNA hybridization (dDDH) values by the Genome-to-Genome Distance Calculator with Formula 2 [44], average amino acid identity (AAI) values by the Kostas lab’s online AAI calculator with two-way AAI [45], and percentage of conserved proteins (POCP) [46]. The genome-based phylogenetic tree was reconstructed using the TYGS server [47], with tree inference performed by FastME [48] based on GBDP distances calculated from genome sequences. Branch lengths are scaled according to the GBDP distance formula d5. The almost full-length 16S rRNA gene sequence of strain W6^T^ was used to screen SRA datasets with identities of 99% using the Integrated Microbial Next Generation Sequencing (IMNGS) platform [49].

### Cultivation and phosphonate degradation experiments

To evaluate the phosphonate degradation capacity and methane production of strain W6^T^, inorganic salt medium [50] was used as the base medium in subsequent experiments. All experiments were performed in triplicate, with cultures grown in acid-washed and autoclaved glass serum bottles, and incubated at 30°C with shaking at 120 rpm.

The phosphate-source utilization experiments were designed to assess strain W6^T^ growth on various phosphorus sources including the following treatments: P free (No P), 200 μM K_2_HPO_4_ (Pi), 200 μM MPn, 200 μM 2-aminoethylphosphonic acid (2-AEP) and 200 μM glyphosate (Exp 1 in Supporting Information Table S1). 20 ml of cultures were grown in 125 ml serum bottles sealed with sterile, breathable membranes to allow gas exchange. To monitor cell growth, 0.5 ml subsamples were collected every 48 hours from three parallel bottles during a 12-day incubation using a non-destructive method. The cells were stained with SYBR Green I and then counted using a CytoFLEX flow cytometer.

To test methane production by strain W6^T^ growth on MPn as the sole phosphorus source (Exp 2 in Supporting Information Table S1), a series of treatments were established: Cultures of W6^T^ were inoculated into media containing either P free (No P), 200 μM Pi, or 200 μM MPn. A negative control, consisting of uninoculated MPn medium was also included to account for abiotic methane production. Each treatment was prepared in 125 ml serum bottles containing 20 ml of medium, leaving a 105 ml headspace. Bottles were sealed with butyl rubber stoppers, crimped with aluminum seals, and incubated under the same conditions described above. Methane concentrations were monitored over a 12-day experimental period. During the first 24 hours, samples were taken every 3–6 hours; thereafter, sampling occurred every 2 days. At each time point, three replicate bottles were sacrificed for destructive sampling. Cultures were terminated by adding saturated HgCl_2_ and preserved for methane concentration determination. Dissolved oxygen (DO) concentrations were monitored by PreSens fiber-optic oxygen meter (OXY-10 ST Prototype). The DOP samples were collected by sacrificing an additional set of 3 replicate bottles at each corresponding time point.

To investigate the effect of Pi availability on MPn degradation and methane production, cultures were amended with 200 μM MPn and one of six initial Pi concentrations: 0, 5, 10, 20, 30, or 40 μM (Exp 3 in Supporting Information Table S1). Each treatment was performed in triplicates, and cultures were incubated for 12 days, after which methane concentrations were measured.

### Measurements of methane concentrations

Samples terminated by saturated HgCl_2_ for methane concentration in the headspace were determined using gas chromatography (Agilent 8860) equipped with a column packed with HayeSep Q (80/100 mesh size) and a flame ionization detector (FID). Briefly, 5 ml of headspace gas from each bottle was injected into the gas chromatography at a flow rate of 20 ml min^−1^, using ultrapure helium (He) as the carrier gas. Methane quantification was based on the FID response, with peak areas calibrated against a CH₄ standard curve prepared using five concentrations: 2.02, 4.99, 10.1, 100, and 1000 ppm. Dissolved CH_4_ concentrations were calculated using the Bunsen coefficient, following the method described by Wiesenburg and Guinasso [51].

### Determination of dissolved inorganic phosphorus and dissolved organic phosphorus

Surface water samples from the reservoir were collected for dissolved inorganic phosphorus (DIP) analysis, and culture samples were prepared for DOP measurement. All samples were filtered through 0.2 μm pore size polycarbonate (PC) membranes prior to analysis. DIP concentrations were determined using an AA3 AutoAnalyzer (Bran + Luebbe, Germany). DOP concentrations were calculated as the difference between total dissolved phosphorus (TDP) and DIP, with TDP determined via the acidic potassium persulfate digestion method outlined by Karl and Björkman [52].

### Transcriptome sequencing and data analysis

The transcriptomic analysis samples were also collected to determine the phosphonate degradation capacity by strain W6^T^ (Exp 4 in Supporting Information Table S1). For this, triplicate cultures grown under P free (No P), 200 μM Pi and 200 μM MPn as the sole phosphorus source, and were harvested destructively for transcriptome sequencing on day four of a 12-day incubation. For the MPn-grown treatment, CH_4_ concentration samples were taken accompanying on days 4, 5, 8, and 12. Additionally, 200 μM Pi was added to a subset MPn-grown cultures on the day 4 to investigate the effect of Pi re-supply on methane production. Methane production was monitored over the next 0, 1, 4, and 8 days, and transcriptomic samples were collected on day 5 (i.e. 1 day after Pi addition) to capture the early transcriptional response to Pi addition in MPn-utilizing cultures. Cells were harvested by centrifugation at 8000 g for 20 min at 4°C. RNA extraction was performed using the RNeasy Mini Kit (Qiagen, cat. no. 74104), followed by DNase treatment to remove genomic DNA (Qiagen, cat. no. 79254). Ribosomal RNA was removed using RiboCop rRNA Depletion Kit for Mixed Bacterial Samples (lexogen, USA), and complementary DNA (cDNA) libraries were constructed with the Illumina® Stranded mRNA Prep, Ligation Kit (San Diego, USA). Paired-end Illumina NovaSeq6000 RNA-sequencing data were deposited under the accession number of PRJNA1198483.

Raw reads were quality-checked, and low-quality reads were filtered out. Q20, Q30, and GC content were analyzed for the cleaned data. The remaining reads were aligned to the *Variovorax xiamenensis* W6^T^ reference genome (GenBank accession: CP139038). Transcript abundance was quantified using RSEM (http://deweylab.github.io/RSEM/), which calculated TPM (transcripts per million) based on read counts and gene lengths. Differential expression analysis was conducted using DESeq2 (https://bioconductor.org/packages/release/bioc/html/DESeq2.html), with genes showing |log ₂ (Fold Change)| > 0 and *P*adj < .05 considered differentially expressed. Enrichment analyses for Gene Ontology (GO, http://www.geneontology.org) terms and Kyoto Encyclopedia of Genes and Genomes (KEGG, http://www.genome.jp/kegg/) pathways were performed to identify statistically enriched metabolic pathways.

### *Phn* gene cluster analysis

To investigate the distribution of the *phn* gene cluster, we analyzed 16 671 genomes from the ProGenomes3 Database [53], which encompasses genomes from four major habitats: freshwater, aquatic, soil, and sediment. The *phn* gene cluster was identified by screening genomes specifically for *phnCDEFGHIJKLMNOP*, which are key genes involved in C–P lyase-mediated phosphonate metabolism. Gene identification was performed using HMMER v3.4, leveraging curated HMM profiles [18] and the trusted cutoffs of each individual model. A valid *phn* gene cluster required at least five of these genes within a 16 000 nucleotides window on a single scaffold or contig, ensuring uninterrupted genomic regions. Contigs with missing key components of the C–P lyase system, such as *phnK*, *phnL*, or *phnM* were excluded. Genomes meeting these criteria were retained for downstream analysis to explore the habitat distribution and taxonomic patterns of *phn* clusters across the four environments. Samples containing information on geographic locations and environmental types were extracted and plotted on the global map using MATLAB. The “geoshow” function was employed to visualize the data and adjust the color shades. A phylogenetic tree was constructed using 120 concatenated single-copy marker genes identified across bacterial genomes containing the *phn* gene cluster. The GTDB-Tk v1.3.0 pipeline [54] with default settings and the GTDB R05-RS95 database [55] were used to identify conserved proteins (120 bacterial proteins) and generate concatenated multi-protein alignments. The phylogenomic tree was constructed using iqtree [56], employing the substitution model Q.yeast+F + I + R10 selected based on model testing [57].

## Results

### Growth and methane production by strain W6^T^ in the presence of methylphosphonate

**Figure 1:**
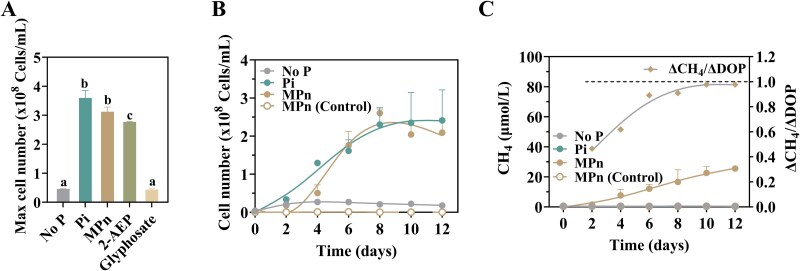
Growth and methane production by strain W6^T^. (A) Growth performance of strain W6^T^ under different phosphorus sources as the sole P source (Exp 1 in supporting information Table S1). P free (No P), K_2_HPO_4_ (Pi), MPn, 2-aminoethylphosphonic acid (2-AEP). Cell numbers represent the maximum values observed over a 12-day incubation period, with measurements taken every 48 hours. (B) Comparative growth dynamics of strain W6^T^ in media supplemented with pi and MPn as the sole phosphorus sources (Exp 2 in supporting information Table S1). (C) Methane production by strain W6^T^ and correlation between net methane production and DOP consumption of strain W6^T^ in the presence of MPn (Exp 2 in supporting information Table S1).

To explore the physiological adaptations of bacteria to P limitation and MPn metabolism under aerobic conditions, we successfully isolated a strain named W6^T^ from a subtropical P-limited (dissolved inorganic phosphate concentration: 0.27 ± 0.16 μmol/L) reservoir in Xiamen, China. This isolate serves as a valuable model for understanding microbial strategies in P-scarce environments. Growth was negligible in P-free media, underscoring the essential phosphorus requirement in sustaining cellular proliferation. Strain W6^T^ showed robust growth in media containing 2-AEP or MPn but failed to grow with the anthropogenic phosphonate glyphosate (Fig. 1A). Despite a prolonged lag phase in MPn-supplemented media, the final cell density match that of Pi-supplemented cultures, indicating that MPn can effectively support cells’ growth (Fig. 1B).

MPn degradation by strain W6^T^ was accompanied by CH_4_ production (Fig. 1C). Notably, CH_4_ accumulation was absent in axenic controls containing MPn alone, confirming its origin from bacterial metabolism in oxic conditions (Supporting Information Fig. S1A). Over a 12-day culture period, strain W6^T^ continuously produced CH_4_, with an average production rate of 92 nmol L^−1^ h^−1^. Even when the cells were in a lag period (initial 24-hour phase), methane production was at a rate of 4.0 nmol L^−1^ h^−1^ (**Supporting Information**  Fig. S1B). This metabolic capability and activity of strain W6^T^ suggests that MPn turnover in natural systems is probably rapid, thus preventing MPn accumulation and complicating its detection in oxic, P-limited aquatic systems.

MPn, a phosphonate with a stable C–P bond, contains a phosphate and a methyl group. Its metabolism as a P source requires MPn uptake, dealkylation, and subsequent CH_4_ release. Mass balance analysis over the culture period showed that 25.7 ± 6.3 μM of DOP was consumed, accompanied with 25.5 ± 1.0 μM CH_4_ production. This near-stoichiometric relationship highlights the efficiency of MPn utilization by strain W6^T^ for both cellular growth and methane release. During the lag phase of growth in MPn-supplemented media, the net CH_4_ production-to-DOP consumption ratio (ΔCH_4_/ΔDOP) was initial low (0.4) but increased to approximately 1 as the culture approached the stationary phase (Fig. 1C). This observation suggests that dealkylation—the cleavage of the stable C–P bond to release CH_4_ and liberate phosphorus—may represent a rate-limiting step in the metabolic pathway. The prolonged lag phase observed in MPn-supplemented media compared to Pi-supplemented media highlights the time requirement for metabolic adaptation to dealkylation pathway.

### Phosphate regulation of microbial methane production

**Figure 2:**
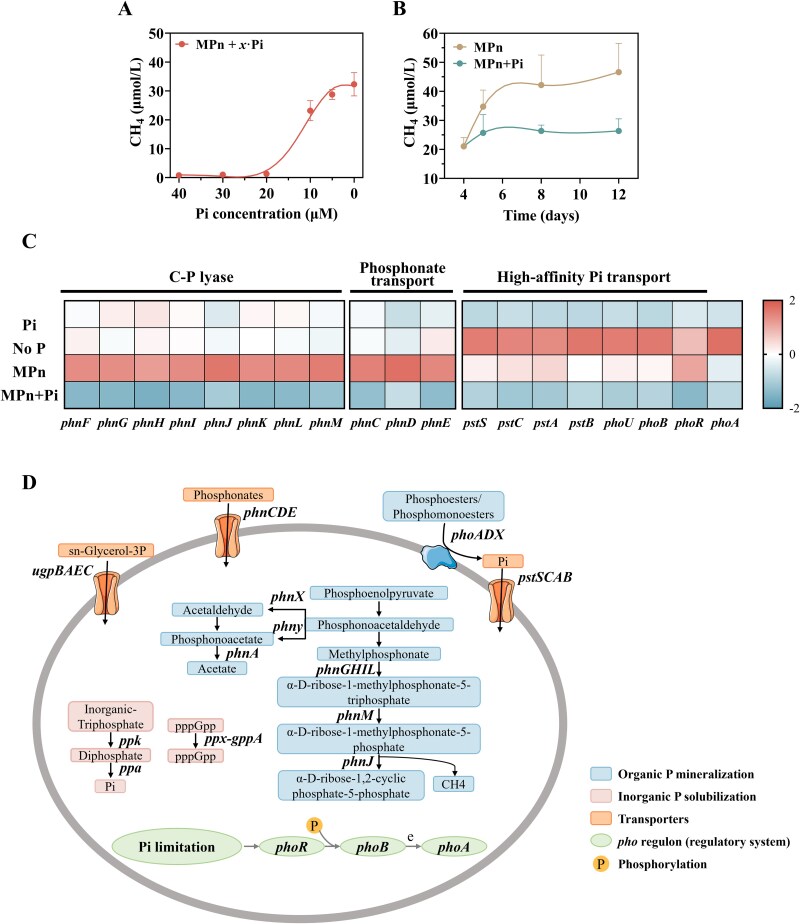
Effect of phosphate concentration on methane production by strain W6^T^. (A) Methane production response of strain W6^T^ cultured with 200 μM MPn and varying concentrations of Pi (0 to 40 μM) (Exp 3 in supporting information Table S1). (B) Methane production by strain W6^T^ under MPn and MPn +Pi conditions (Exp 4 in supporting information Table S1). All cultures were initially incubated with 200 μM MPn for 4 days. On day 4, 200 μM Pi was added to one set of MPn-grown cultures (MPn + Pi). Methane production was monitored for 8 days following Pi addition (up to day 12). The MPn group received no Pi and served as the control. (C) Transcriptomic responses of P acquisition-related genes in strain W6^T^ of four treatments (Exp 4 in supporting information Table S1). Pi is phosphate, No P stands for no phosphorus, MPn is methylphosphonate, MPn +Pi represents strain W6^T^ cultured with MPn for 4 days followed by the addition of 200 μM Pi. Transcriptome samples were collected on day 4 for No P, Pi, and MPn treatments, and on day 5 (i.e. 1 day after Pi addition) for MPn + Pi. Color bar represents the Z-score normalized gene expression (TPM) level. (D) Genes encoding P cycle-related functions, which were identified in strain W6^T^ genome.

To examine how phosphate availability regulates microbial methane production, strain W6^T^ was cultured with 200 μM MPn and simultaneously supplement with varying concentrations of Pi ranging from 0 to 40 μM. Results revealed a striking trend: CH_4_ production from MPn increased substantially as Pi concentration decreased, peaking when Pi was nearly absent (Fig. 2A). Conversely, higher Pi concentrations repressed CH_4_ production, indicating that Pi scarcity strongly induces the MPn degradation pathway, resulting in elevated CH_4_ emissions.

To further evaluate this regulation, 200 μM Pi was added to W6^T^ cultures pre-grown with MPn for 96 hours. The results showed that within 24 hours of Pi addition, CH_4_ production decreased significantly (t-test, *P* < .05), persisting over the extended observation period (Fig. 2B). The decline reflects a metabolic shift of strain W6^T^ towards utilizing the more energetically favorable Pi over MPn.

Transcriptomic analyses under four different phosphorus conditions—No P, Pi, MPn, and MPn + Pi—provide molecular insights into this regulation. Among the 4663 identified differentially expressed genes (DEGs), a substantial proportion exhibited pronounced transcriptional shifts (Supporting Information Table S2, Supplementary Dataset S1). Gene Ontology (GO) categorization showed that these DEGs were predominantly enriched in catalytic activity, cellular anatomical entity, binding, cellular process and metabolic process (Supporting Information Fig. S2A). KEGG pathways analysis revealed that most DEGs were involved in pathways related to membrane transport, carbohydrate metabolism, amino acid metabolism and cellular community (Supporting Information Fig. S2B). Compared to Pi conditions, genes associated with the high-affinity phosphate transport system (*pstABCS*), the phosphate starvation response transcription activator *phoB*, and alkaline phosphatase *phoA* were significantly upregulated in No P treatment (Fig. 2C**,**  Supplementary Dataset S1). Interestingly, these genes, which serve as markers for Pi deficiency, did not return to the expression levels observed under Pi conditions in the MPn treatment group, indicating reliance on alternative phosphorus acquisition strategy. This is particularly intriguing, as it contrasts with the typical regulation observed in other organisms, where both the high-affinity phosphate transport system and the C–P lyase pathway are generally co-regulated under phosphate starvation as part of the *pho* regulon [58], with activation driven by Pi scarcity. Furthermore, when comparing the transcriptomes of W6^T^ grown in MPn versus normal Pi conditions, genes related to phosphonate transport (*phnC-E*) were highly upregulated in the presence of MPn, and the *phn* gene cluster (*phnG-M*) for C–P lyase was also upregulated (Fig. 2C), which is critical for the biodegradation of MPn to release CH_4_ and P for cell growth. Notably, the gene *phnJ*, crucial for C–P bond cleavage, was repressed upon Pi addition (Supplementary Dataset S1), halting methane production. This indicates that when Pi is available, strain W6^T^ prioritizes its utilization, shutting down the energy-intensive MPn degradation pathway. This finding aligns with the hypothesis proposed by Teikari et al. [59], which suggests that the expression of the C–P lyase pathway is not solely regulated by Pi scarcity, but requires the presence of a suitable phosphonate substrate such as MPn.

### Strain W6^T^ belongs to a novel species of the genus *Variovorax*

Given its role as both a phosphorus recycler and methane producer under phosphorus-limited conditions, strain W6^T^’s taxonomic identity was further examined to understand its ecological and evolutionary significance. Nearly complete 16S rRNA gene sequences of strain W6^T^ (1437 bp; GenBank accession number OR790634) was used for similarity-based searches against the taxonomically united 16S rRNA database in EzBioCloud. Strain W6^T^ shared its highest similarity with *Variovorax gossypii* JM-310^T^ (99.15%), followed by *Variovorax beijingensis* 502^T^ (99.01%) and *Variovorax guangxiensis* DSM 27352^T^ (98.96%). Phylogenetic analyses using Maximum Likelihood (ML, Supporting Information Fig. S3), Minimum Evolution (ME, Supporting Information Fig. S4) and Neighbor-Joining (NJ, Supporting Information Fig. S5) algorithms consistently clustered strain W6^T^ with members of the genus *Variovorax* and formed a separated branch within the genus.

**Figure 3:**
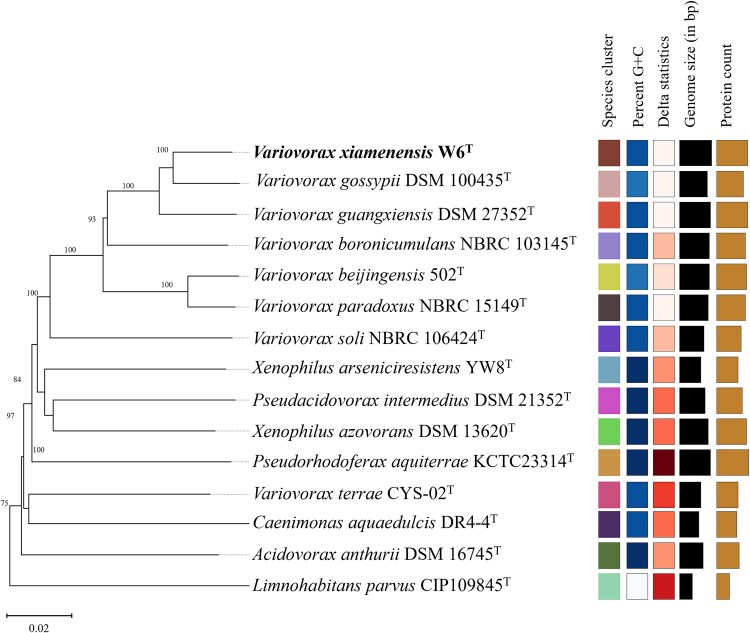
Phylogenetic tree inferred with FastME from GBDP distances calculated from genome, showing the positions of strain W6^T^ and type species affiliated with the family *Comamonadaceae*. The numbers above branches are GBDP pseudo-bootstrap support values >60% from 100 replications. Bar, 0.02 substitutions per nucleotide position. Legend to the squares is shown in the right-hand side columns. Species cluster: Strains of the same species cluster are indicated with the same color. Percent G + C content: a difference of >1% indicates a potentially unreliable identification result since the G + C content should not vary by more than 1% within species when computed from genome sequences. Delta statistics: Δ values, overall tree likeness. Genome size: overall genome sequence length. Protein counts: Number of proteins detected by the server.

To further refine the taxonomic placement within the genus *Variovorax*, the complete genome of W6^T^ (GenBank accession number CP139038) was sequenced. The genomic analysis revealed a circularized genome of 7 201 473 bp with 6514 coding sequences (CDSs) and a DNA G + C content of 67.6%. The phylogenomic analysis (Fig. 3) positioned strain W6^T^ within a monophyletic clade alongside *Variovorax gossypii* JM-310^T^ and *Variovorax guangxiensis* DSM 27352^T^. However, genomic comparison indicated that strain W6^T^ falls below the thresholds for species discrimination, < 94.0% average nucleotide identity (ANI), < 52.0% dDDH, < 94.0% AAI, and < 86.0% POCP (Supporting Information Table S3). These values were markedly lower than the standard thresholds for ANI (95%) and dDDH (70%) in bacterial species differentiation [60, 61], while they exceeded the recommended cut-off values for AAI (70%) and POCP (50%) in bacterial genera delimitation [46, 62]. Thus, these values confirm that strain W6^T^ is novel at the species level while remaining within the *Variovorax* genus.

Distinctive phenotypic characteristics between strain W6^T^ and related species of the genera *Variovorax* (Supporting Information Table S4) further support the classification of W6^T^ as a novel species. The colony morphology and transmission electron micrograph image of strain W6^T^ are shown at Supporting Information Fig. S6. The polar lipid profile includes diphosphatidylglycerol (DPG), phosphatidylglycerol (PG), phosphatidylcholine (PE), and several unidentified phospholipids (PL) and glycolipids (APL) (**Supporting Information**  Fig. S5). The fatty acid results compared with other species are listed in Supporting Information Table S5. Therefore, based on the results of polyphasic analyses performed in this study, strain W6^T^ represents a new member of the genus *Variovorax*, for which the name *Variovorax xiamenensis* sp. nov. is proposed.

### Global distribution and ecological significance of *Variovorax xiamenensis* sp. nov.

**Figure 4:**
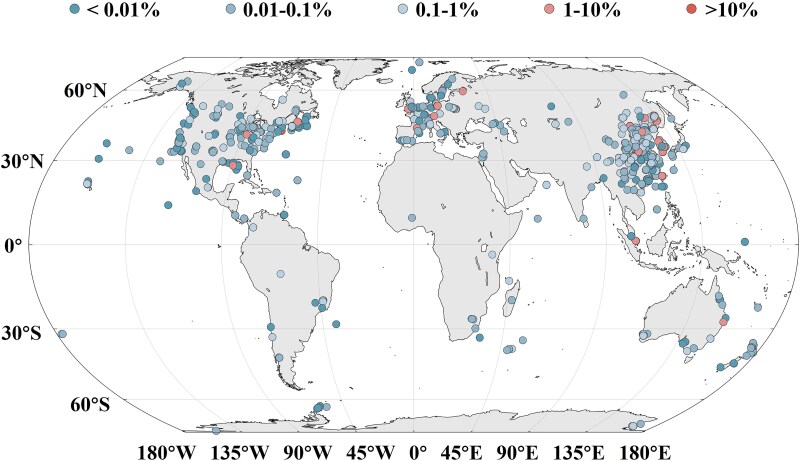
Global distribution of phylotypes closely related to *Variovorax xiamenensis* W6^T^ based on 16S rRNA gene sequences (≥ 99% identity) identified in public SRA datasets. While these hits cannot be confidently assigned to the same species or strain, they suggest a broad presence of closely related *Variovorax xiamenensis* lineages in natural environments.

To gain insight into the potential ecological significance and global distribution of *Variovorax xiamenensis* sp. nov., the near full-length 16S rRNA gene sequence of strain W6^T^ was used to query publicly available Short Read Archive (SRA) datasets. Hits with ≥99% identity were considered as phylotypes closely related to *V. xiamenensis* sp. nov. These phylotypes were detected across diverse natural environments, including freshwater, groundwater, seawater, and soil, with notably high relative abundances (up to 19.7%) in groundwater and freshwater (Fig. 4**,**  Supplementary Dataset S2). While this analysis does not allow species- or strain-level resolution, it suggests that members of the *V. xiamenensis* lineage or its close relatives may be ecologically widespread. This broad distribution could reflect shared genomic traits such as the complete C–P lyase pathway (*phnC*-*E*, *phnG*-*M*) and high-affinity phosphate transport systems (*pstS*, *pstC*, *pstA*, *pstB*), which confer adaptability to phosphorus-limited environments on a global scale.

### *Pseudomonadota* as major contributors to the *phn* gene cluster

**Figure 5:**
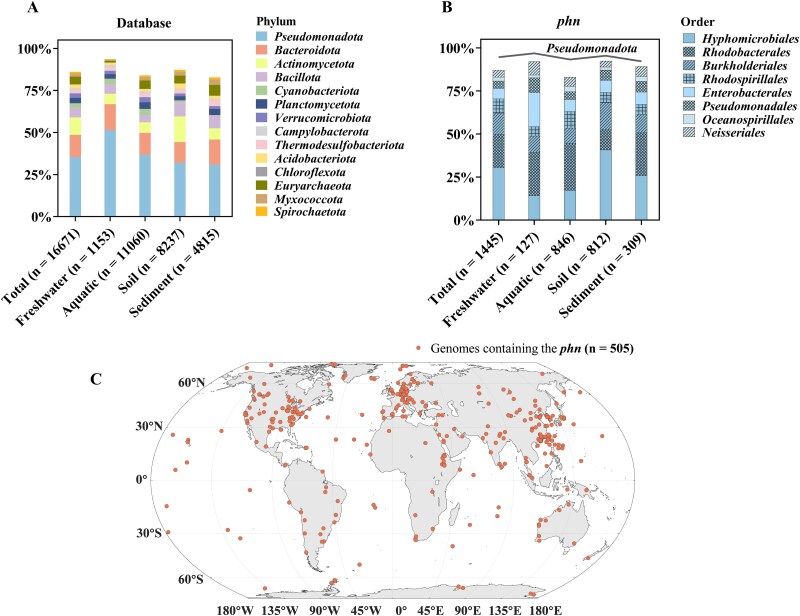
Taxonomic composition and geographic distribution of genomes containing the *phn* gene cluster. (A) Phylum-level classification of genomes in the ProGenomes3 database across four habitats: freshwater, aquatic, sediment, and soil. (B) Order-level classification of genomes containing the *phn* gene cluster. The *Pseudomonadota* phylum accounts for over 90% of genomes harboring the *phn* gene cluster. The y-axis shows the percentage composition, excluding taxonomic groups contributing less than 1% of the total. The x-axis represents the total number of genomes and their distribution across habitats. (C) Geographic distribution of genomes containing the *phn* gene cluster (*n* = 505). Only genomes with reliable geographic metadata are displayed, representing 505 out of the total 1445 genomes. Many genomes were excluded due to missing or inaccurate geographic information.

To investigate the prevalence and ecological role of the *phn* gene cluster, we performed a comprehensive analysis of over 16 000 prokaryotic genomes from diverse habitats, including freshwater, aquatic, sediment, and soil environments [53]. The *phn* gene cluster, comprising the phosphonate transporter complex (*phnC*-*E*) and the multi-subunit C–P lyase complex (*phnG*-*M*), is critical for cleaving carbon-phosphorus (C–P) bonds. Our results show that approximately 9% (1445 of 16 671) of the analyzed genomes possess intact *phn* gene cluster (Fig. 5, Supplementary Dataset S3), highlighting a widespread genetic potential for C–P bond utilization among prokaryotes. Notably, genomes derived from freshwater habitats show a slightly higher prevalence, with around 11% (127 of 1153) encoding the complete *phn* cluster, indicating an adaptive response to phosphorus limitation commonly observed in freshwater ecosystems (Supporting Information Table S6).

The taxonomic distribution of the *phn* gene cluster revealed its presence in more than 100 bacterial families across 7 distinct phyla (Supporting Information Fig. S8), underscoring its evolutionary persistence and ecological importance. However, the distribution is disproportionately dominated by certain taxa (Fig. 5B): over 95% of genomes identified with the *phn* gene cluster belong to the phylum *Pseudomonadota*. In contrast, its occurrence in other groups, such as *Cyanobacteria*, is only around 2%. Within *Pseudomonadota*, approximately 85% of genomes containing the *phn* cluster are concentrated in six primary orders: *Hyphomicrobiales*, *Rhodobacterales*, *Burkholderiales*, *Rhodospirillales*, *Pseudomonadales*, and *Enterobacterales*. The dominance of *phn* gene cluster within *Pseudomonadota* genomes is not solely a consequence of the phylum’s representation in sequencing data (Fig. 5A). Instead, it reflects their ecological specialization in utilizing MPn as an alternative phosphorus source. This metabolic capability likely provides an advantageous adaptation in P-limited systems, reinforcing the potential ecological significance of *Pseudomonadota* in global phosphorus cycling and methane production.

## Discussion

### Phosphonate metabolism as an adaptive strategy to phosphorus scarcity

This study highlights the crucial role of phosphonate metabolism as an adaptive mechanism for microbial survival in P-depleted environments. Climate models predict that global warming will intensify thermal stratification in aquatic systems, reducing nutrient mixing between surface and deep waters, and exacerbating P scarcity in many freshwater and marine environments [4, 63]. The prevalence of phosphonate utilization under P-limited conditions is corroborated by studies in diverse aquatic systems. For example, in Brazilian soda lakes, the abundance of *phnJ* gene sequences, a marker gene for phosphonate catabolism, strongly correlates with declining orthophosphate concentrations [64]. These patterns reflect the ecological significance of phosphonate metabolism as a critical microbial strategy for maintaining growth in P-depleted waters. In oligotrophic marine systems, phosphonate metabolism has been estimated to supply on average 11% of the phosphorous required for sustaining primary production [65], further underscoring the importance of this pathway in nutrient-poor environments. Our findings demonstrate that *Variovorax xiamenensis* sp. nov., represented by strain W6^T^, is well adapted to such conditions, with the capability to utilize MPn and 2-AEP as sole phosphorous sources under laboratory conditions.

Beyond individual metabolic advantages, phosphonate utilization profoundly shapes nutrient dynamics and microbial community interactions. *Cyanobacteria*, for instance, recruit heterotrophic phosphonate-degrading bacteria to exploit this resource and sustain growth under phosphate-depleted conditions [66]. Similarly, mutualistic relationships observed in sponge-associated microbiomes and macroalgal microbiota highlight the ecological importance of phosphonate cycling in nutrient-limited marine regions such as the Mediterranean and Sargasso Seas [67, 68]. These interactions suggest that phosphonate catabolism is not merely a microbial survival strategy but also a key driver of nutrient recycling, contributing to the resilience and functioning of aquatic ecosystems under nutrient stress.

### Methylphosphonate catabolism and oxic methane production

Our study further underscores the pivotal role of MPn catabolism in oxic CH_4_ production (OMP), particularly in P-limited aquatic ecosystems [22, 23, 50]. MPn degradation represents an alternative CH₄ production pathway, distinct from classical methanogenesis associated with anoxic sediments. Recent estimates from the western tropical North Atlantic suggest average potential net methane production rates of 0.4 nmol L^−1^ d^−1^ through MPn degradation [65]. Additionally, experimental evidence from two contrasting stratified lakes highlights the significant contribution of MPn to CH₄ supersaturation in oxic surface layers [69].

Our findings align with these observations, as strain W6^T^ demonstrated sustained methane emissions during growth on MPn. Importantly, incubation experiments and transcriptomic analyses revealed that methane production was significantly reduced upon orthophosphate (Pi) supplementation (Fig. 2B), indicating that Pi availability regulates the expression of *phn* genes (Fig. 2C) and controls methane emissions from MPn degradation. This Pi-dependent repression is consistent with regulatory patterns observed in both freshwater and oligotrophic marine systems. For example, Yao et al. [27] reported that methane production by pure bacterial isolates from Lake Matano—a phosphorus-limited tropical lake—was linearly inhibited by increasing Pi concentrations, with complete suppression observed above ~30 μM. Extending beyond pure cultures, Ye et al. [70] conducted in situ Pi amendment experiments in oligotrophic seawaters of the western North Pacific, where CH₄ production from MPn decreased significantly in the presence of Pi. Taken together, these findings point to a regulatory mechanism underscores the dynamic interplay between nutrient availability and microbial methane production, with potential implications for global methane budgets. The ecological and climatic significance of this pathway lies in its potential feedback effects under warming scenarios. Climate-induced stratification and nutrient depletion are likely to expand the ecological niche for MPn-utilizing microbes, increasing OMP and methane emissions from oxic waters. This feedback loop-where warming exacerbates P limitation, enhancing methane production and further contributing to greenhouse gas accumulation—represents a critical yet underappreciated component of aquatic methane cycling.

Despite its documented importance in lakes and marine systems, methane production under oxic conditions is often excluded from models of aquatic methane cycling, which predominantly focus on anoxia, primary production, and temperature dynamics [71, 72]. Incorporating OMP, with explicit consideration of phosphonate metabolism and P availability, is essential for more accurate predictions of methane fluxes in freshwater and marine systems.

### Comparative insights from marine and freshwater systems

While MPn metabolism has been extensively studied in marine environment [20, 73, 74], its role in freshwater systems remains largely underexplored [23, 27–29]. Marine studies have demonstrated that MPn degradation plays an important role in phosphorus cycling and OMP, particularly in oligotrophic regions [22, 24, 65]. However, the presence of MPn in freshwater ecosystems is typically absent or found in low concentrations [75, 76]. Strikingly, our genomic survey reveals a widespread distribution of the *phn* gene cluster in freshwater microbial genomes, suggesting the potential for MPn-driven methane production in freshwater systems. These findings point to the existence of efficient MPn scavenging mechanisms in such environments. Future research should focus on quantifying MPn concentrations in freshwater environments and exploring its role in both phosphorus cycling and methane emissions.

## Conclusion

We demonstrate that *Variovorax xiamenensis* W6^T^, a newly identified species, is a robust example of microbial adaptation to P scarcity, using MPn as both a nutrient source and a substrate for methane production under laboratory conditions. Transcriptomic evidence indicates a nutrient-regulated methane production mechanism, highlighting the intricate connections between microbial metabolism, nutrient availability, and greenhouse gas emissions. Around 9% of prokaryotic genomes possess the *phn* gene cluster, with a notable predominance in *Pseudomonadota*, suggesting their ecological advantage in aquatic environments. However, the lack of direct evidence for MPn in freshwater ecosystems highlights a significant knowledge gap. Future studies should focus on quantifying MPn concentrations in freshwater systems and exploring its role in phosphorus cycling and methane emissions. By integrating insights from marine and freshwater environments, we can develop a more comprehensive understanding of the global significance of phosphonate metabolism in aquatic ecosystems.

### Description of *Variovorax xiamenensis* sp. nov.

*Variovorax xiamenensis* (xia.men.en’is. N.L. masc. Adj. xiamenensis pertaining to Xiamen, a district in Fujian, PR China, where the type strain was isolated).

Colonies are pale yellow, convex, uniformly circular and approximately 1 mm in diameter when grown on LB medium. Cells are Gram-negative, aerobic, non-flagellated and rod-shaped (0.5–0.8 μm wide and 1.0–1.5 μm long). Growth occurs at 15–40°C (optimum 25–35°C), at pH 5.0–9.0 (optimum 7.0–8.0) and in 0–4% (w/v) NaCl (optimum 0–2%). Both oxidase and catalase reactions are positive. Strain W6^T^ exhibits positive enzyme activities for Alkaline phosphatase, Esterase (C4), Esterase lipase (C8), Lipase (C14), Leucine arylamidase, Valine arylamidase, Cystine arylamidase, Trypsin, Acid phosphatase, naphtol-AS-Bl-phosphoamidase, *α*-glucosidase, *β*-glucosidase and *α*-fucosidase. Positive for nitrate reduction but negative for nitrite reduction. Esculin and urea are hydrolyzed. The predominant ubiquinone is Q-8. The major fatty acids are C_16:0_, C_17:0_ cyclo and summed feature 3 (C_16:1_*ω*7*c*/C_16:1_*ω*6*c*). The major polar lipids contain DPG, PG, phosphatidylmonomethylethanolamine and PE. The DNA G + C content calculated from the whole genome sequence is 67.6%.

The type strain W6^T^ (= MCCC 1K08691^T^ = KCTC 8209^T^) was isolated from surface water of substropical reservoir in Xiamen, China. The 16S rRNA and genome sequences are submitted to GenBank under accession numbers OR790634 and CP139038, respectively.

## Supplementary Material

Supplemental_Material_Revised_v2_ycaf100

Supplementary_Dataset_S1_ycaf100

Supplementary_Dataset_S2_ycaf100

Supplementary_Dataset_S3_ycaf100

## Data Availability

The 16S rRNA and genome sequences have been deposited in GenBank under accession numbers OR790634 and CP139038, respectively. Transcriptomic sequencing data generated in this study are available in the NCBI database under BioProject number PRJNA1198483. Source data supporting the findings of this study are provided with this publication.
