# Supplementary material for: Phosphonate-driven oxic CH4 production by Variovorax xiamenensis sp. nov.: insights from marine and freshwater microbial adaptations to P-limitation
Source: ISME Commun. 2025 Jun 16;5(1):ycaf100. doi: 10.1093/ismeco/ycaf100 (PMC12226003; doi:10.1093/ismeco/ycaf100)
Supplement: Supplemental_Material_Revised_v2_ycaf100 [file supplemental_material_revised_v2_ycaf100.pdf]

Phosphonate-Driven Oxic CH_4 Production by *Variovorax xiamenensis* sp. nov.: Insights from Marine and Freshwater Microbial Adaptations to P-Limitation

Yu Wang[1], Shaohe Wang[1], Zifu Xu[2], Silin Ni[2], Min Nina Xu[2*], Shuh-Ji Kao[2*]

[1]State Key Laboratory of Marine Environmental Science & College of Ocean and Earth Sciences, Xiamen University, Xiamen, China

[2]State Key Laboratory of Marine Resource Utilization in South China Sea, School of Marine Science and Engineering, Hainan University, Haikou, China

*Corresponding authors. Min Xu, College of Ocean Science and Engineering, Hainan University, No. 58 Renmin Road, Haidian Island, Meilan District, Haikou, Hainan 570228, China. E-mail: minxu@hainanu.edu.cn; Shuh-Ji Kao, College of Ocean Science and Engineering, Hainan University, No. 58 Renmin Road, Haidian Island, Meilan District, Haikou, Hainan 570228, China. E-mail: sjkao@hainanu.edu.cn

Contents of this file:

Supplementary Figures: 1–8

Supplementary Tables: 1-6

Supplementary Figures

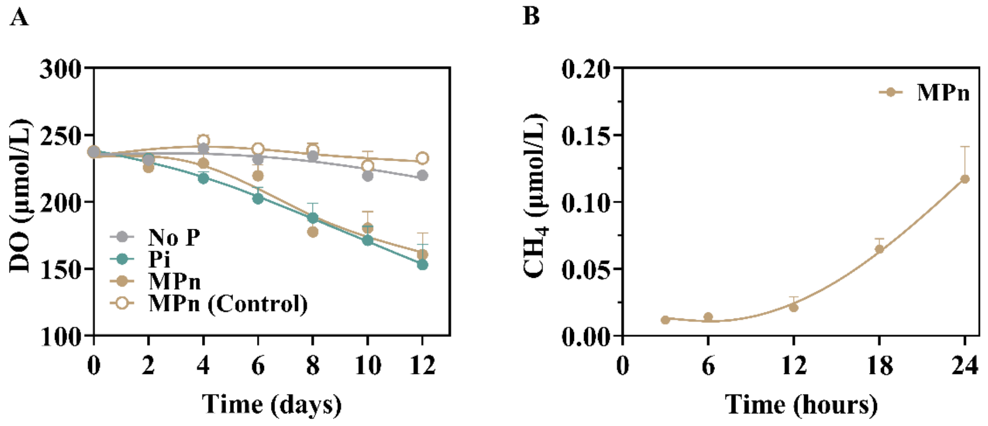

Supplementary Fig. S1 A Oxygen consumption of strain W6^T of three phosphorous treatments. MPn (Control): the treatment group without adding strain W6^T culture served as negative control. **B** Methane release from MPn degradation over a 24h period.

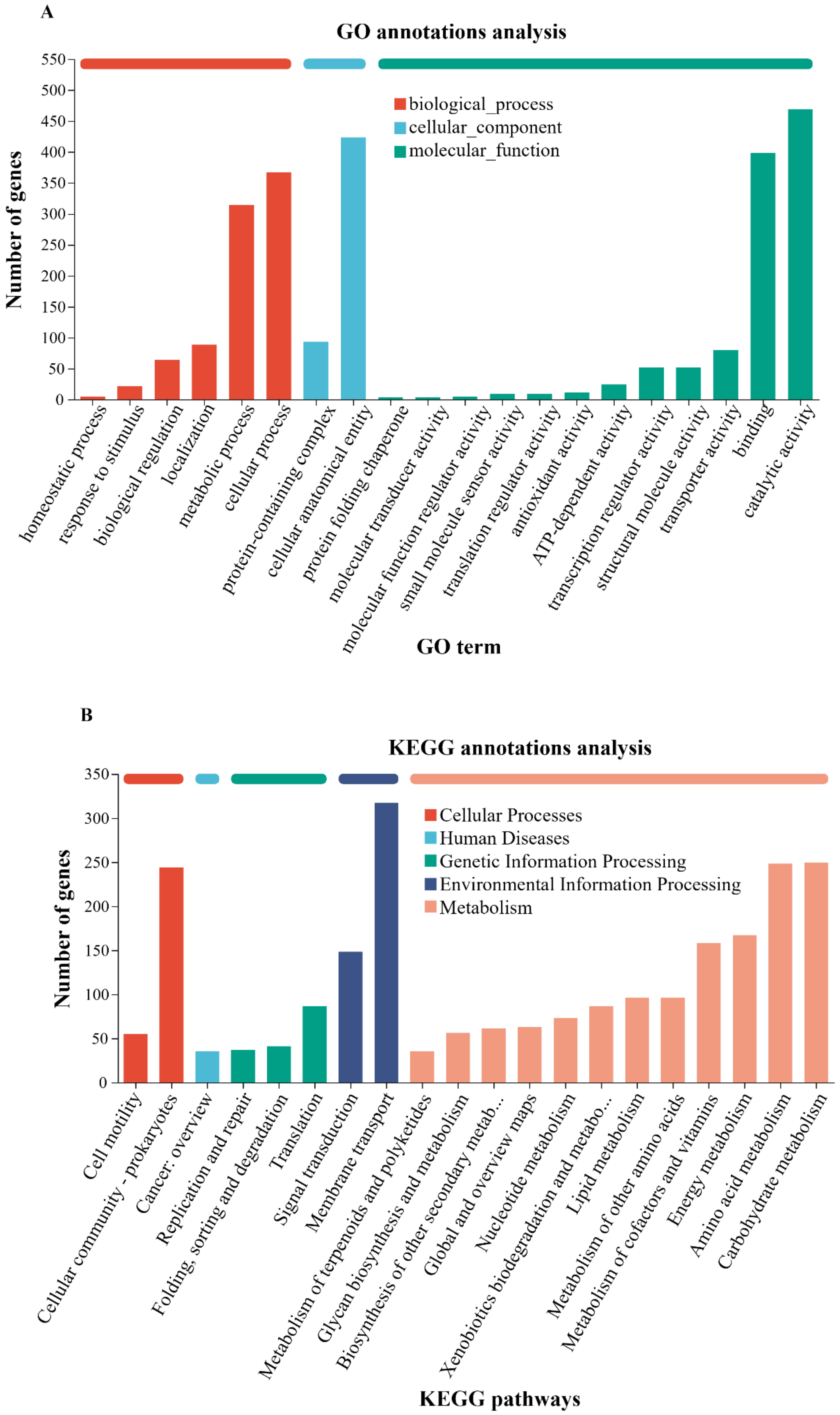
A
GO annotations analysis
Number of genes
550
500
450
400
350
300
250
200
150
100
50
0
biological_process
cellular_component
molecular_function
homeostatic process
response to stimulus
biological regulation
localization
metabolic process
cellular process
protein-containing complex
cellular anatomical entity
protein folding chaperone
molecular transducer activity
molecular function regulator activity
small molecule sensor activity
translation regulator activity
antioxidant activity
ATP-dependent activity
transcription regulator activity
structural molecule activity
transporter activity
binding
catalytic activity
GO term
B
KEGG annotations analysis
Number of genes
350
300
250
200
150
100
50
0
Cellular Processes
Human Diseases
Genetic Information Processing
Environmental Information Processing
Metabolism
Cell motility
Cellular community - prokaryotes
Cancer: overview
Replication and repair
Folding, sorting and degradation
Translation
Signal transduction
Membrane transport
Metabolism of terpenoids and polyketides
Glycan biosynthesis and metabolism
Biosynthesis of other secondary metab...
Global and overview maps
Nucleotide metabolism
Xenobiotics biodegradation and metabo...
Lipid metabolism
Metabolism of other amino acids
Metabolism of cofactors and vitamins
Energy metabolism
Amino acid metabolism
Carbohydrate metabolism
KEGG pathways

Supplementary Fig. S2 A GO analysis classified the DEGs into three groups (molecular function, biological process and cellular component). **B** KEGG metabolic pathway annotation map.

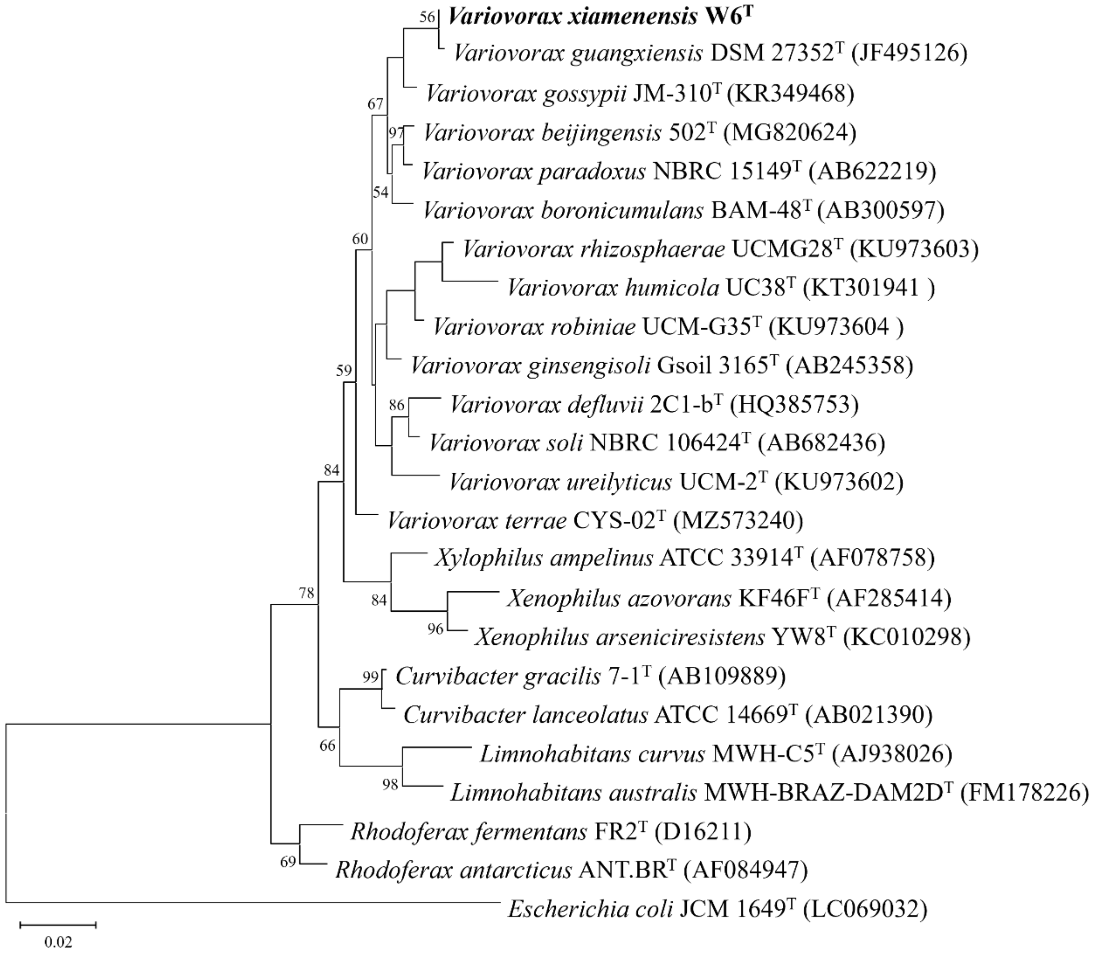

Supplementary Fig. S3 Maximum-Likelihood tree showing the phylogenetic positions of strain W6^T and related members in the family *Comamonadaceae*, based on 16S rRNA gene sequences. *Variovorax xiamenensis* W6^T was marked bold. Bootstrap values (expressed as percentages of 1,000 replications) > 60% are shown at branch nodes. Bar, 0.02 substitutions per nucleotide position. *Escherichia coli* JCM 1649^T (LC069032) was used as outgroup.

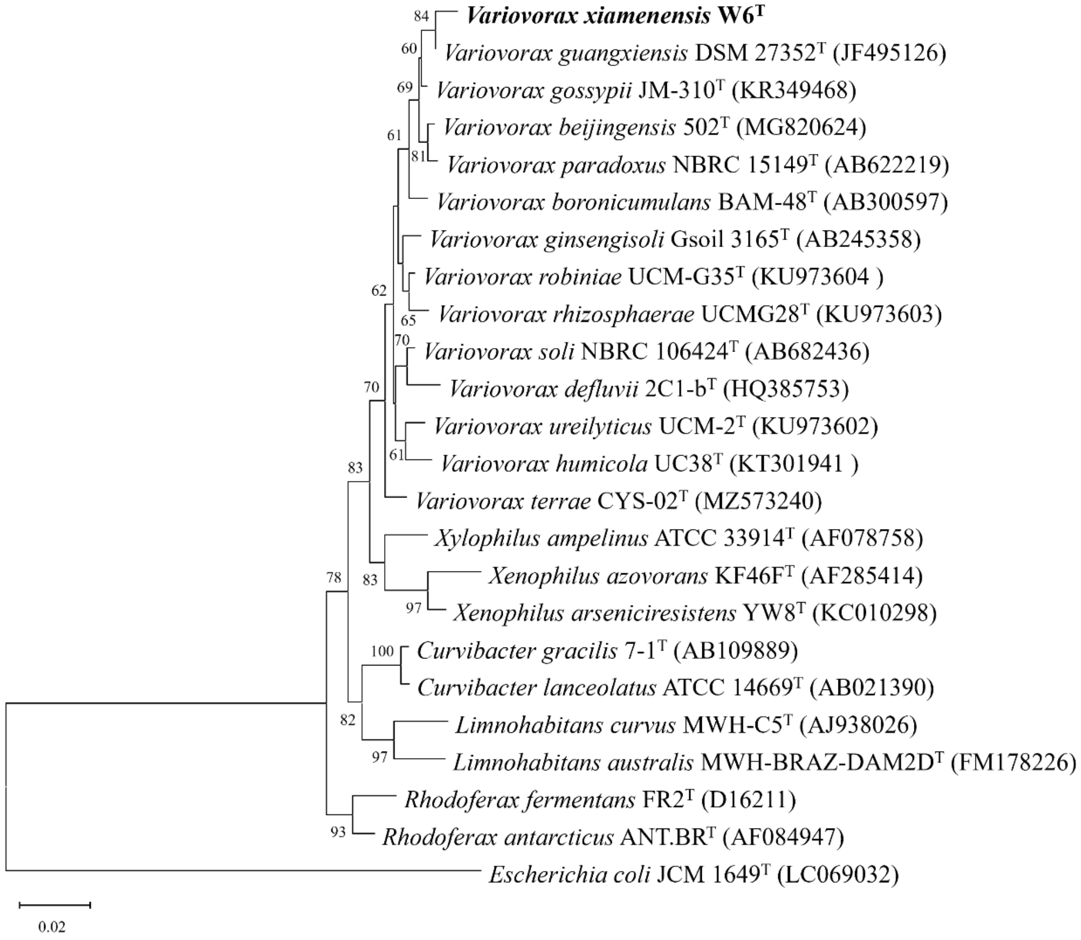

Supplementary Fig. S4 Minimum Evolution tree showing the phylogenetic positions of strain W6^T and related members in the family *Comamonadaceae*, based on 16S rRNA gene sequences. *Variovorax xiamenensis* W6^T was marked bold. Bootstrap values (expressed as percentages of 1,000 replications) > 60% are shown at branch nodes. Bar, 0.02 substitutions per nucleotide position. *Escherichia coli* JCM 1649^T (LC069032) was used as outgroup.

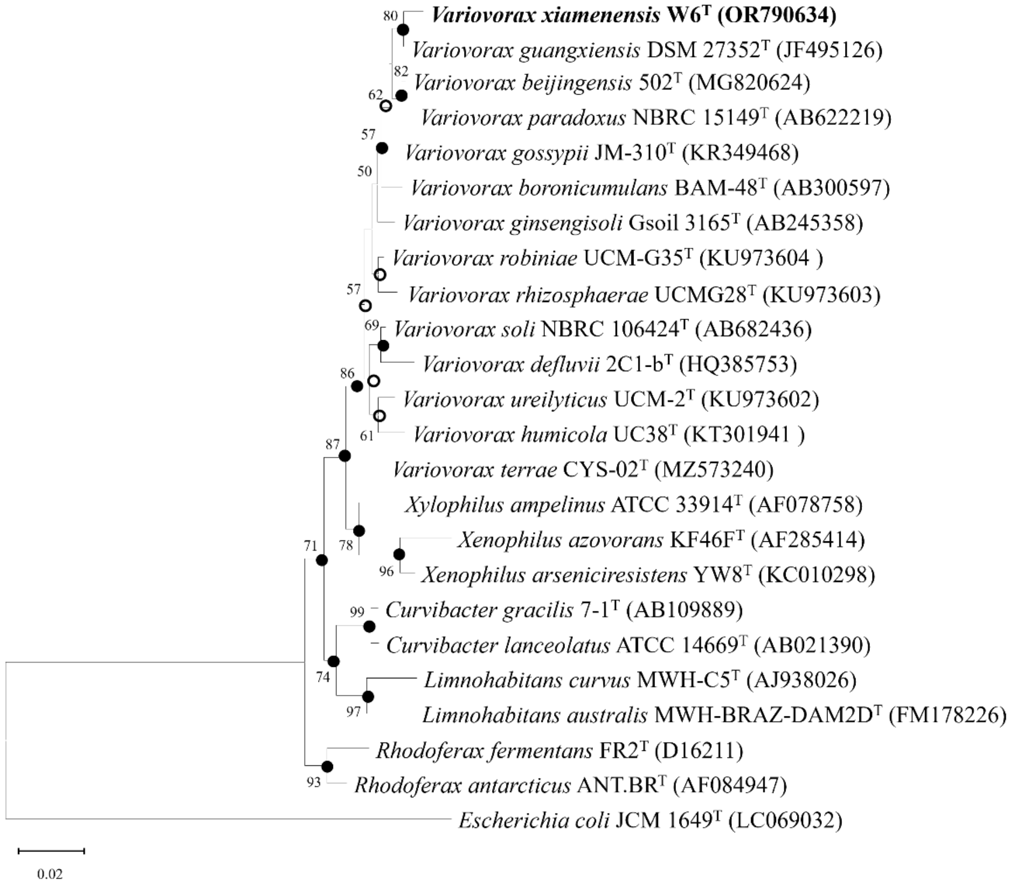

Supplementary Fig. S5 Neighbour-joining tree showing the phylogenetic positions of strain W6^{T} and related members in the family *Comamonadaceae* based on nearly complete 16S rRNA gene sequences. *Variovorax xiamenensis* W6^{T} is marked with bold text. Open circles indicate nodes that were recovered in the minimum-evolution trees. Filled circles indicate nodes that were also recovered in the maximum-likelihood and minimum-evolution trees for the same sequences. Bootstrap values (expressed as percentages of 1000 replications) > 60% are shown at branch nodes. Bar, 0.02 substitutions per nucleotide position. *Escherichia coli* JCM 1649^{T} (LC069032) was used as outgroup.

A B

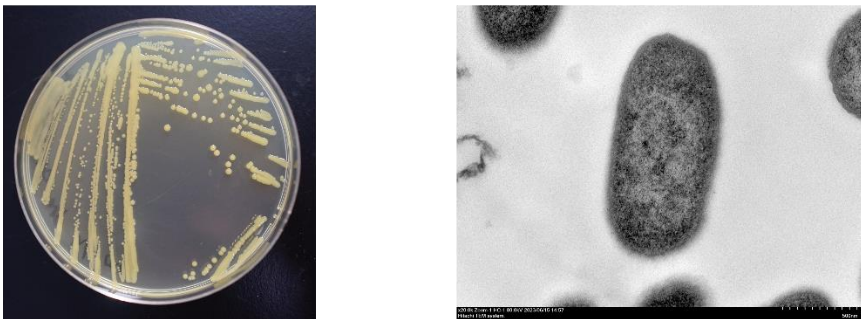

Supplementary Fig. S6 A The morphological characteristics of the colony. **B** Transmission electron micrograph image of strain W6^T cultured in LB at 30 °C for 36 hours.

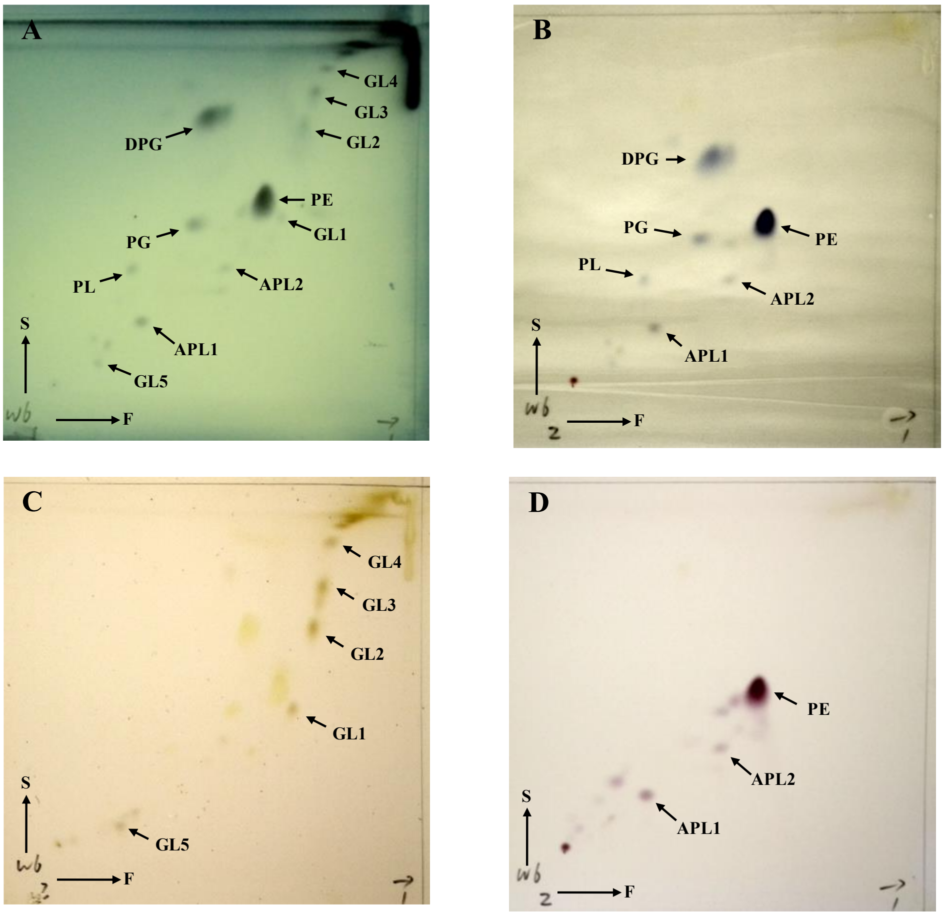

Supplementary Fig. S7 Two-dimensional TLCs of polar lipids of strain W6^T which were stained with molybdophosphoric acid **A**, Zinzadze reagent **B**, α-naphthol reagent **C** and ninhydrin reagent **D**, respectively. DPG, diphosphatidylglycerol; PE, Phosphatidylethanolamine; PG, Phosphatidylglycerol; PL, phospholipid; APL, aminophospholipids; GL, glycolipid.

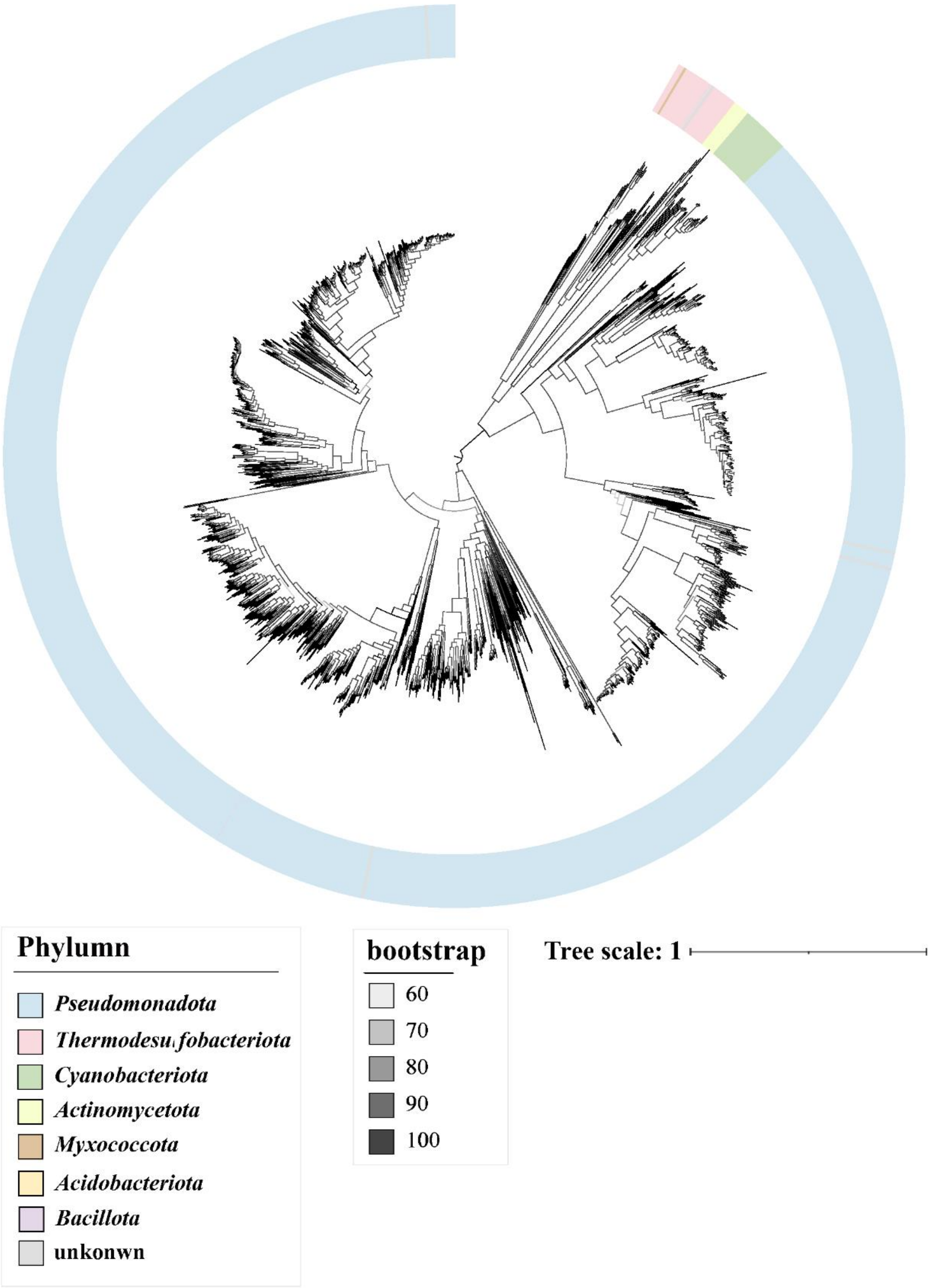

Supplementary Fig. S8 The phylogenetic tree is constructed from 120 concatenated single-copy marker genes across 1,445 bacterial genomes identified as carrying a *phn* gene cluster.

Supplementary Tables

Table S1 Summary of experimental design for phosphonate degradation and methane production by strain W6^T.

Experiment	Treatment conditions	Duration	Sampling method	Measured products
Exp 1. Phosphate-source utilization	No P<br>200 μM Pi<br>200 μM MPn<br>200 μM 2-AEP<br>200 μM Glyphosate	12 days	Subsampling	Cell density (48 h intervals)
Exp 2. Methane production on MPn as sole P source	No P	12 days	Destructive sampling (At each time point, 3 replicate bottles were sacrificed for sampling: first for cell density measurement, followed by termination with $HgCl_2$, and then methane and DO measurements. If DOP was measured, an additional set of three replicate bottles was sacrificed for each corresponding time point.)	Cell density, Methane, DO (48 h intervals)
	200 μM Pi			
	200 μM MPn			Cell density, DO, DOP (48 h intervals), Methane (every 3–6 h (days 0–1), then every 2 days)
	200 μM MPn (Control,without strain W6^T inoculation)			Cell density, Methane, DO (48 h intervals)
Exp 3. Pi gradient addition to MPn cultures	MPn (200 μM) + Pi (0, 5, 10, 20, 30, 40 μM)	12 days	Destructive sampling: 3 replicate bottles were terminated on day 12 for methane concentration determination	Methane
Exp 4. Transcriptomic analysis and Pi re-supply	No P<br>200 μM Pi	12 days	Destructive sampling: 3 replicate bottles on day 4 for RNA-seq	Transcriptome (RNA-seq)
	200 μM MPn		Destructive sampling: 3 replicate bottles on day 4 for RNA-seq and 3 replicate bottles were terminated at each time point for methane concentration determination	Transcriptome (RNA-seq), Methane (day 4, 5, 8, 12)
	200 μM Pi added to MPn cultures on day 4		Destructive sampling: 3 replicate bottles on day 5 (1 day after Pi addition) for RNA-seq and 3 replicate bottles were terminated at each time point for methane concentration determination	

Table S2 Numbers of differentially expressed genes when comparing stain W6^T cultured under four different phosphorous treatments.

Comparison group	Up-regulated	Down-regulated	Total
MPn vs Pi	2	63	65
No P vs Pi	203	53	256
MPn+Pi vs MPn	2901	1679	4580

Table S3 Average nucleotide identity (ANI), digital DNA-DNA hybridization (dDDH), average amino acid identity (AAI) and percentage of conserved proteins (POCP) differences between the genome sequence of W6^T and its closest relative.

Strains	ANI (%)	dDDH (%)	AAI (%)	POCP (%)
Variovorax gossypii JM-310^T	93.30	51.10	93.27	78.59
Variovorax guangxiensis DSM 27352^T	92.34	47.10	93.98	85.52
Variovorax beijingensis 502^T	85.75	41.00	84.09	73.07
Variovorax paradoxus NBRC 15149^T	85.91	41.50	84.68	73.24
Variovorax boronicumulans BAM-48^T	86.93	31.80	85.51	77.78
Variovorax ginsengisoli Gsoil 3165^T	80.75	23.70	75.49	58.76
Variovorax soli NBRC 106424^T	80.28	23.50	73.78	62.44

Table S4 Differential characteristics of strain W6^T and the type strains of closely related *Variovorax* species.

Characteristic	W6^T	*Variovorax beijingensis* 502^T	*Variovorax boronicumulans* BAM-48^T	*Variovorax paradoxus* ATCC 17713^T
Growth at (°C)	15-40	20-40 [a]	4-37 [b]	4-40 [c]
Growth at pH	5.0-9.0	5.0-9.0 [a]	5.0-9.0 [b]	6.0-9.0 [c]
NaCl tolerance (%)	0-4.0	0-2.0 [a]	0-1.0 [b]	0-2.0 [c]
Catalase	+	+	+	+
Nitrate Reduction	w	+	-	+
Hydrolysis of Urease	-	+	+	-
Gelatin	-	-	+	-
Aesculin	+	+	+	+
Major fatty acids (> 10%)	$C_{16:0}$, $C_{17:0}$ cyclo and summed feature 3	$C_{10:0}$ 3-OH, $C_{16:0}$, $C_{17:0}$ cyclo and summed feature 3 [a]	$C_{10:0}$ 3-OH, $C_{12:0}$, $C_{14:0}$ 2-OH, summed feature 3[a]	$C_{10:0}$ 3-OH, $C_{16:0}$, $C_{17:0}$ cyclo and summed feature 3 [a]
DNA G+C content (%)	67.6	67.4 [a]	71.2 [b]	67.0 [c]

Summed Features represent groups of two fatty acids that could not be separated by gas-liquid chromatography with the MIDI system. Summed feature 3 comprised $C_{16:1}$ *ω7c*/ $C_{16:1}$ *ω6c*. +, positive; -, negative; w, weakly reaction. Data from this study unless indicated otherwise.

a. Data from [1].

b. Data from [2].

c. Data from [3].

Table S5 Comparison of fatty acid compositions (%) of strain W6^T and the type strains of related *Variovorax* species.

Strains: 1, W6^T; 2, *Variovorax gossypii* JM-310^T (data from [4]); 3, *Variovorax guangxiensis* DSM 27352^T (data from [5]); 4, *Variovorax beijingensis* 502^T (data from [1]); 5, *Variovorax paradoxus* DSM 30034^T (data from [1]); 6, *Variovorax boronicumulans* BAM-48^T (data from [1]).

Fatty acids	1	2	3	4	5	6
$C_{8:0}$ 3-OH	-	-	-	-	4.7	-
$C_{9:0}$ 3-OH	-	-	-	-	2.1	-
$C_{10:0}$	-	-	-	2.8	-	-
$C_{10:0}$ 3-OH	3.97	12.8	18.42	**26.2**	15.6	**52.4**
$C_{12:0}$	3.5	3.7	4.85	8.1	8.7	10.3
$C_{12:0}$ 3-OH	-	-	-	-	-	-
$C_{14:0}$	2.72	-	-	1.6	1.4	-
$C_{14:0}$ 2-OH	1.72	2.5	3.14	6.2	7.9	11.0
$C_{15:1}$ *ω6c*	-	-	-	-	1.4	-
$C_{16:0}$	**35.37**	**27.7**	**20.33**	**12.9**	**15.1**	5.6
$C_{16:1}$ 2-OH	-	-	2.34	2.2	1.5	1.8
$C_{16:1}$ *ω5c*	-	-	-	-	1.2	-
$C_{17:0}$	-	-	-	-	-	1.2
$C_{17:0}$ cyclo	**22.2**	**3.9**	**18.92**	**14.5**	**17.5**	4.9
iso-$C_{17:0}$ 3-OH	-	-	-		1.3	-
$C_{18:0}$	1.06	-	-	-	-	-
$C_{18:1}$*ω7c*	-	-	12.26	-	-	**13.1**
$C_{18:1}$ 2-OH	-	-	1.23	-	-	-
Summed Features*						
3	**17.68**	-	13.93	**21.4**	**17.4**	**10.0**
4	-	**29.8**	-	-	-	-
7	-	**19.6**	-	-	-	-
8	9.01	-	-	5.5	4.9	1.7

*Summed Features represent groups of two fatty acids that could not be separated by gas-liquid chromatography with the MIDI system. Summed feature 3 comprised $C_{16:1}$ *ω7c*/ $C_{16:1}$ *ω6c*; summed feature 4 comprised $C_{16:1}$ ω7c/ iso-$C_{15:0}$ 2-OH; summed feature 7 comprised $C_{18:1}$ ω7c/ ω9t/ ω12t and summed feature 8 comprised $C_{18:1}$ *ω7c*/ $C_{18:1}$ *ω6c*. -, Less than 1 % or not detected. Major fatty acids (> 10%) are highlighted in bold.

Table S6 Prevalence of *phn* gene clusters in different habitats based on the progenomes3 database.

Habitat Classification	Total	*phn*	%
Freshwater	1153	127	11.01%
Aquatic	11060	846	7.65%
Sediment	4815	309	6.42%
Soil	8237	812	9.86%
Total	16671	1445	8.67%

Supplementary References

1. Gao JL, Sun YC, Xue J, Sun PB, Yan H, Khan MS *et al.* *Variovorax beijingensis* sp. nov., a novel plant-associated bacterial species with plant growth-promoting potential isolated from different geographic regions of beijing, china. *Syst Appl Microbiol*. 2020;**43**:126135 https://doi.org/10.1016/j.syapm.2020.126135
2. Miwa H, Ahmed I, Yoon J, Yokota A, Fujiwara T. *Variovorax boronicumulans* sp nov., a boron-accumulating bacterium isolated from soil. *Int J Syst Evol Micr*. 2008;**58**:286-289 https://doi.org/10.1099/ijs.0.65315-0
3. Nguyen TM, Kim J. Description of *variovorax humicola* sp nov., isolated from a forest topsoil. *Int J Syst Evol Micr*. 2016;**66**:2520-2527 https://doi.org/10.1099/ijsem.0.001082
4. Kämpfer P, Busse HJ, McInroy JA, Glaeser SP. *Variovorax gossypii* sp. nov., isolated from gossypium hirsutum. *Int J Syst Evol Micr*. 2015;**65**:4335-40 https://doi.org/10.1099/ijsem.0.000581
5. Gao JL, Yuan M, Wang XM, Qiu TL, Li JW, Liu HC *et al.* *Variovorax guangxiensis* sp nov., an aerobic, 1-aminocyclopropane-1-carboxylate deaminase producing bacterium isolated from banana rhizosphere. *Antonie Van Leeuwenhoek*. 2015;**107**:65-72 https://doi.org/10.1007/s10482-014-0304-3
